# Pregnancy Outcomes of Assisted Reproductive Technology (ART) Cycle Complicated by Ovarian Hyperstimulation Syndrome (OHSS): Case Series Study

**DOI:** 10.7759/cureus.42303

**Published:** 2023-07-22

**Authors:** Samaher Alfaraj, Ashwaq A Alharbi, Hind J Aldabal, Yara S Alhabib, Shihanah AlKhelaiwi

**Affiliations:** 1 Obstetrics and Gynecology, Ministry of National Guard Health Affairs (MNGHA), Riyadh, SAU; 2 Reproductive Endocrinology and Infertility, King Fahad Medical City (KFMC), Riyadh, SAU; 3 Medicine, King Saud bin Abdulaziz University for Health Sciences, Riyadh, SAU; 4 Medicine and Surgery, King Saud bin Abdulaziz University for Health Sciences, Riyadh, SAU

**Keywords:** king abdulaziz medical city, case series, pregnancy outcomes, ovarian hyperstimulation syndrome, assisted reproductive technology

## Abstract

Background: Ovarian hyperstimulation syndrome (OHSS) is a frequent, potentially lethal side effect of assisted reproductive technology (ART), distinguished by symptoms such as ovarian enlargement, ascites, and pleural effusion.

Objective: This study is designed to study the effect of assisted reproductive technology (ART) cycle complicated by OHSS on pregnancy outcomes.

Method: A case series study at King Abdulaziz Medical City (KAMC) in Riyadh, Saudi Arabia, was executed to examine the pregnancy outcomes in in vitro fertilization (IVF) and intracytoplasmic sperm injection (ICSI) cycles. Fifteen patients were admitted to the IVF unit between January 2015 and December 2021. Data were retrieved from patients' medical records, and descriptive statistical methods were employed to analyze participants' data.

Results: The study assessed pregnancy outcomes for 15 female participants (mean age=31.1 years, SD=3.46) with a BMI range of 20-40 (mean BMI=29.6, SD=6.4), of whom 33.3% were classified as obese. The primary factor of infertility was anovulation (66.7%), followed by male factors (20%). About 26.7% of those affected by OHSS had moderate OHSS, and 73.3% had severe OHSS, with 100% of those with severe OHSS having undergone three embryo transfers. None of the participants developed gestational diabetes mellitus (DM), but one participant had high blood sugar levels (6.67% of total participants), with a mean glucose of 6.3±2.0. There were no instances of preeclampsia, gestational hypertension, abnormal placentas, or congenital abnormalities in newborns among the participants. Preterm deliveries were common, with 33.3% delivering between 32 and 37 weeks, 6.7% before 28 weeks, and 33.3% within 28-32 weeks. Overall, 73.3% of the participants experienced pregnancy, and the birth mode was almost evenly split between vaginal and cesarean birth.

Conclusion: In conclusion, this research provides an exploration into the outcomes of pregnancies in women undergoing assisted reproductive technology treatments complicated by ovarian hyperstimulation syndrome. It shows anovulation as a prevalent cause of infertility and a noteworthy incidence of severe OHSS. Despite these challenges, a significant number of women were able to experience pregnancy, although preterm deliveries and abortions were common. The delivery methods were fairly balanced between vaginal birth and cesarean section. These findings underscore the necessity for more effective strategies to manage OHSS and improve pregnancy outcomes in ART procedures.

## Introduction

Assisted reproductive technology (ART) represents a significant stride in infertility management, offering newfound hope to countless couples worldwide facing difficulties in conceiving naturally [1]. The ART process involves several steps designed to circumvent fertility barriers and promote the successful fertilization and implantation of the embryo. These steps typically include ovarian stimulation to produce multiple eggs, egg retrieval, fertilization in a laboratory setting (via in vitro fertilization or intracytoplasmic sperm injection), and subsequent embryo transfer to the woman's uterus. Each stage is carefully monitored and controlled to optimize the likelihood of a successful pregnancy. This collection of procedures showcases how ART effectively addresses diverse fertility challenges, allowing more couples the opportunity to experience parenthood [2]. Despite the success rates of ART, it is not free of complications, and one of the most common adverse effects is ovarian hyperstimulation syndrome (OHSS) [3].

OHSS is a potentially life-threatening complication characterized by ovarian enlargement, ascites, and pleural effusion, among other symptoms [4]. OHSS can occur in 1%-14% of ART cycles [5], and its severity can range from mild to severe, with severe OHSS being a rare but serious complication that requires hospitalization and intensive care management [6]. OHSS is a rare yet severe complication of ART that necessitates hospitalization and intensive care management [6]. It can manifest in mild to severe forms, with complications ranging from abdominal discomfort and bloating to serious health risks such as ovarian enlargement, severe abdominal pain, rapid weight gain, difficulty breathing, and imbalances in blood electrolytes. In severe cases, it may lead to life-threatening conditions such as ovarian torsion, acute respiratory distress syndrome, and thromboembolic events. Thus, the management of OHSS is critical in safeguarding the health and well-being of patients undergoing ART [6].

The precise pathophysiology of ovarian hyperstimulation syndrome (OHSS) remains incompletely understood, although it is generally attributed to an overstimulation of the ovaries by gonadotropins [7]. In more detail, the excessive ovarian stimulation is thought to trigger a surge in the release of vasoactive substances such as vascular endothelial growth factor (VEGF). This, in turn, enhances vascular permeability, causing fluid to shift from the intravascular compartment into the third space, which includes the peritoneal cavity and pleural space. This fluid shift can lead to complications like ascites, pleural effusion, hypovolemia, electrolyte imbalances, and in severe cases, critical conditions such as organ dysfunction and thromboembolism. While this theory provides a basis for understanding OHSS, additional research is required to fully elucidate the pathophysiology and to develop effective prevention and treatment strategies [7].

OHSS can have significant consequences for both the mother and the fetus, including preterm labor, thromboembolic events, and fetal growth restriction [8]. Several studies have investigated the impact of OHSS on pregnancy outcomes, but the results have been inconsistent [9]. Some studies have reported no significant differences in pregnancy rates between cycles complicated by OHSS and cycles without OHSS [10].

Several studies have highlighted lower pregnancy rates, increased risks of miscarriage, and higher instances of preterm delivery in ART cycles complicated by OHSS [11]. Interpretation of these findings, however, can be complicated due to differences in factors such as study design, sample size, and the severity of OHSS observed among participants. Furthermore, different studies highlighted the cultural contexts as significant contributors to these outcomes. Variations in healthcare access, societal perspectives on infertility, prevalence of certain risk factors, as well as adoption and adherence to ART procedures may differ across cultures and geographical regions. These factors, alongside the inherent differences in study methodologies, could account for the diversity seen in the reported outcomes across different studies [11]. Therefore, there is a need for further research to clarify the impact of OHSS on pregnancy outcomes. This study aims to investigate the pregnancy outcomes of ART cycles complicated by OHSS.

## Materials and methods

This case series study was conducted at King Abdulaziz Medical City (KAMC) in Riyadh, Saudi Arabia. KAMC is a healthcare facility affiliated with King Abdullah International Medical Research Center (KAIMRC). The study was performed in the In-Vitro Fertilization (IVF) unit at KAMC, which had fully equipped clinics and procedure rooms with experienced physicians and ultrasonographers.

The study participants included all patients admitted to the IVF unit from January 2015 to December 2021. The inclusion criteria for this study encompassed patients aged 18 to 45 years who not only underwent an in vitro fertilization (IVF) or intracytoplasmic sperm injection (ICSI) cycle and had a positive human chorionic gonadotropin (hCG) test following embryo transfer but also presented with symptoms or a diagnosis of ovarian hyperstimulation syndrome (OHSS). Thus, the focus of this research is on women who experienced both a positive pregnancy outcome following IVF/ICSI and complications arising from OHSS. The exclusion criteria were patients with missing data or loss of follow-up, those who used frozen sperm, those who had a biochemical pregnancy, frozen embryo transfer, preimplantation genetic testing (PGT), known parental chromosomal abnormalities, Mullerian duct abnormalities (uterine malformations), known cases of diabetes type 1 or 2, cervix postoperative conization, and premature ovarian failure.

This study employed a non-probability purposive sampling method within a case series study design. The sample size of 15 was selected based on the availability of eligible cases within the study period and feasibility constraints. Purposive sampling was chosen to ensure the inclusion of patients who satisfied the defined criteria, specifically, women aged 18 to 45 years who had undergone an IVF or ICSI cycle, had a positive hCG test after embryo transfer, and also developed OHSS. Concurrently, this method facilitated the exclusion of those who met the study's exclusion criteria. Each case was selected meticulously, based not only on compliance with the inclusion criteria but also considering the depth and richness of information they could offer to meet the study's objectives. This process guaranteed that all selected cases provided relevant and insightful data concerning the intersection of ART, positive pregnancy outcomes, and OHSS complications.

Data were collected from patient's medical records, including demographic data, clinical characteristics, ART cycle parameters, and pregnancy outcomes (successful pregnancies following assisted reproductive technology procedures, incidence of abortions, prevalence of preterm deliveries, and the mode of delivery, which included vaginal births and cesarean sections). The collected data were analyzed using descriptive statistics, including frequencies, percentages, and means, as appropriate. The findings of this study were presented in tables and graphs and compared with the existing literature on the same topic.

The diagnosis of the severity of ovarian hyperstimulation syndrome (OHSS) is based on specific criteria. These generally include the patient's symptoms, clinical findings, and in some cases, laboratory values. Mild OHSS is characterized by abdominal bloating, mild abdominal pain, and ovaries measuring 5-12 cm. Moderate OHSS involves the symptoms of mild OHSS plus nausea (with or without vomiting), an enlarged ovary (greater than 12 cm), and ultrasound evidence of ascites.

Severe OHSS, on the other hand, presents more seriously with symptoms including severe abdominal pain, decreased urination, shortness of breath, a tendency to thrombosis, marked ovarian enlargement (>12 cm), and substantial ascites or hydrothorax. Laboratory findings may reveal hemoconcentration, coagulation abnormalities, kidney dysfunction, and liver dysfunction. For this study, patients were classified into moderate and severe OHSS based on these standard criteria.

Ethical approval was obtained from the Institutional Review Board at King Abdullah International Medical Research Center (KAIMRC), and informed consent was obtained from all study participants. Confidentiality and anonymity were maintained throughout the study.

Prior to their participation, all study participants were provided with a detailed explanation of the study's purpose, procedures, potential benefits, and risks. This information was conveyed both verbally and through a written document in a language they could understand. Participants were given ample time to ask questions and were reassured that their participation was entirely voluntary, with no impact on their medical care should they choose not to participate. Once they fully understood the study and expressed their willingness to participate, they were asked to sign the informed consent form. For participants who were unable to sign, a thumb impression was obtained as a substitute for a signature. All consents were taken before any data collection or study-specific procedures were initiated, ensuring that participants' rights and autonomy were upheld. Confidentiality and anonymity were maintained throughout the study.

The data collected from the medical records of the study participants were entered into a computerized database using Microsoft Excel (Microsoft Corporation, Redmond, Washington, USA). The database was then exported to SPSS version 27 (IBM Corporation, Chicago, IL, USA) for statistical analysis. Descriptive statistics were used to analyze the participants' data, including frequencies, percentages, means, and standard deviations. The data were organized into tables and graphs for presentation and interpretation. The level of significance for all statistical tests was set at p<0.05. The findings of the data analysis were interpreted in the context of the existing literature on the topic.

## Results

**Figure 1:**
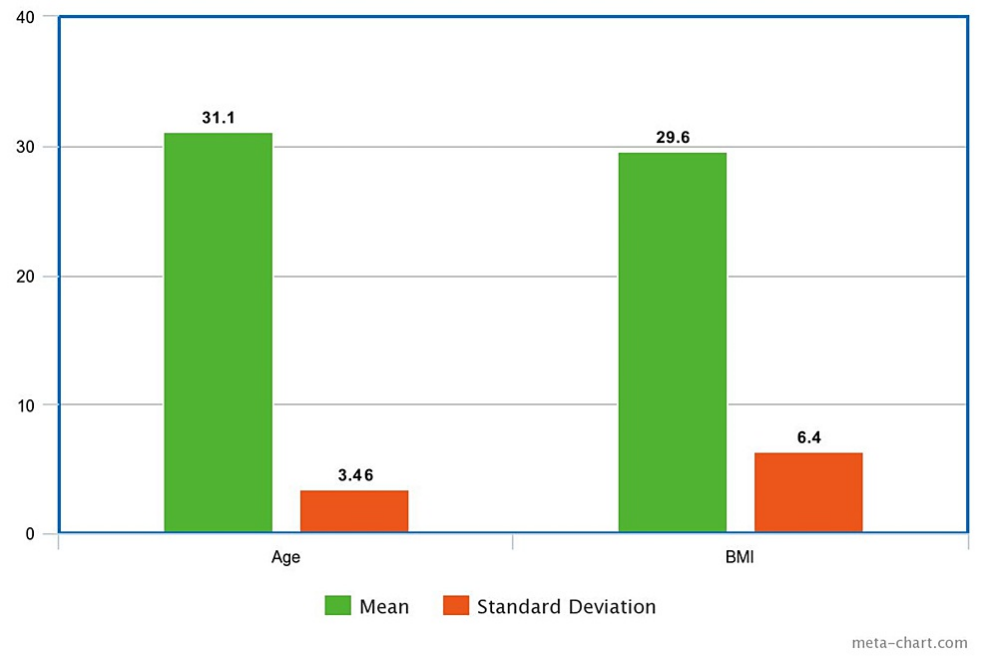
Mean and Standard Deviation for Age and BMI BMI: body mass index.

The research incorporated a group of 15 women participants whose ages spanned between 26 and 36 years. The average age of the participants was found to be 31.1 years, with a standard deviation of 3.46. The body mass index (BMI) of the participants varied from 20 to 40, with an average BMI recorded at 29.6 and a standard deviation of 6.4 (Figure 1).

**Figure 2:**
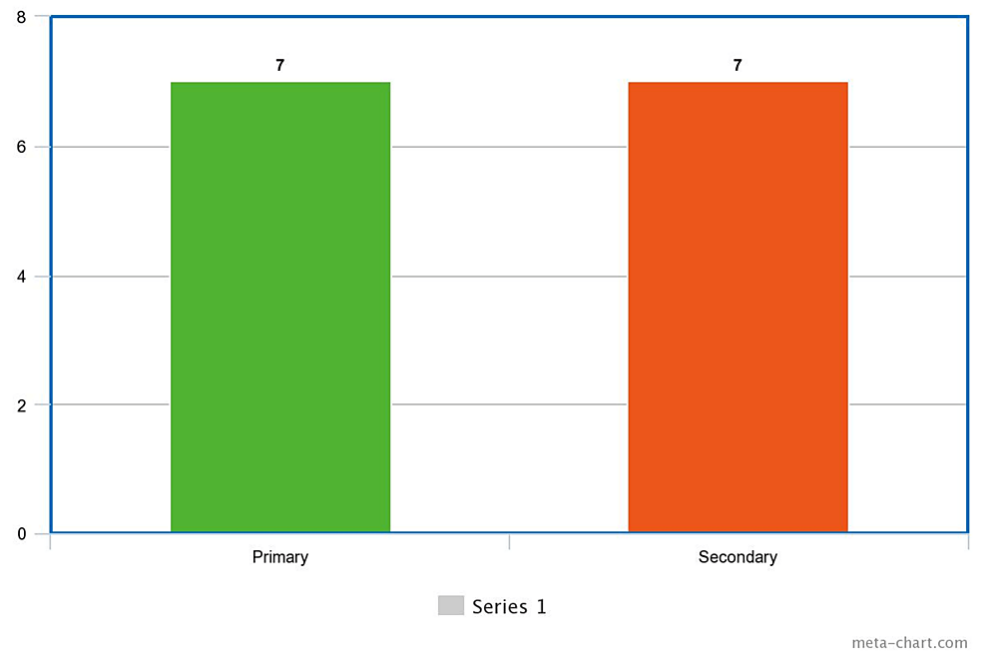
Type of Infertility

**Figure 3:**
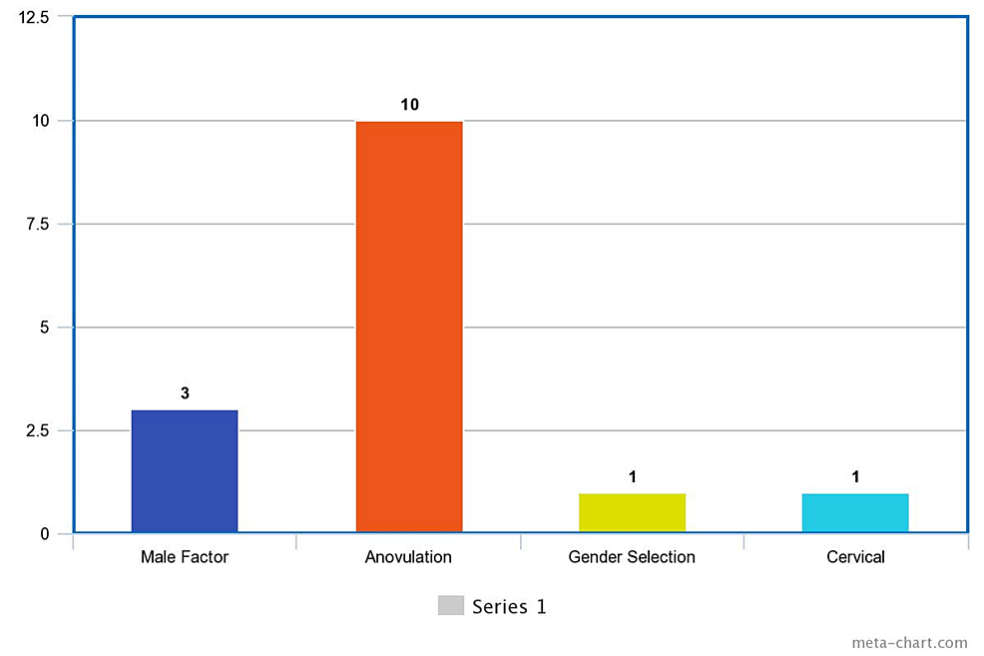
Factors of Infertility

As for the type of infertility they experienced, primary infertility was identified in seven participants and secondary infertility in the other seven (Figure 2). The cause of infertility differed among the women: male factors were identified in three cases, anovulation was identified in 10, gender selection in one, and cervical factor in another case (Figure 3).

**Figure 4:**
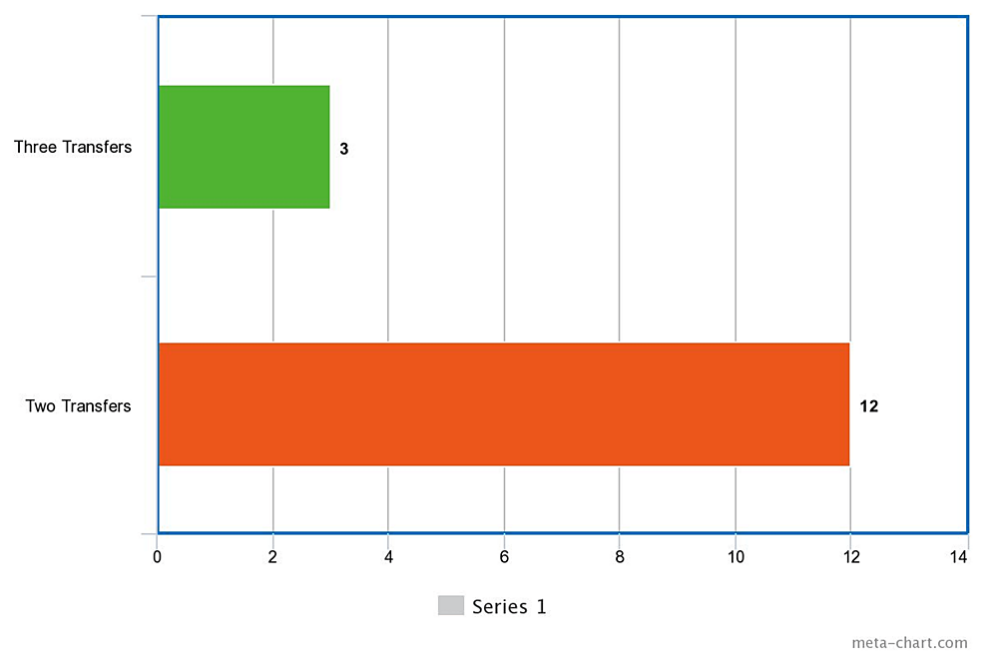
Number of Embryo Transfer

**Figure 5:**
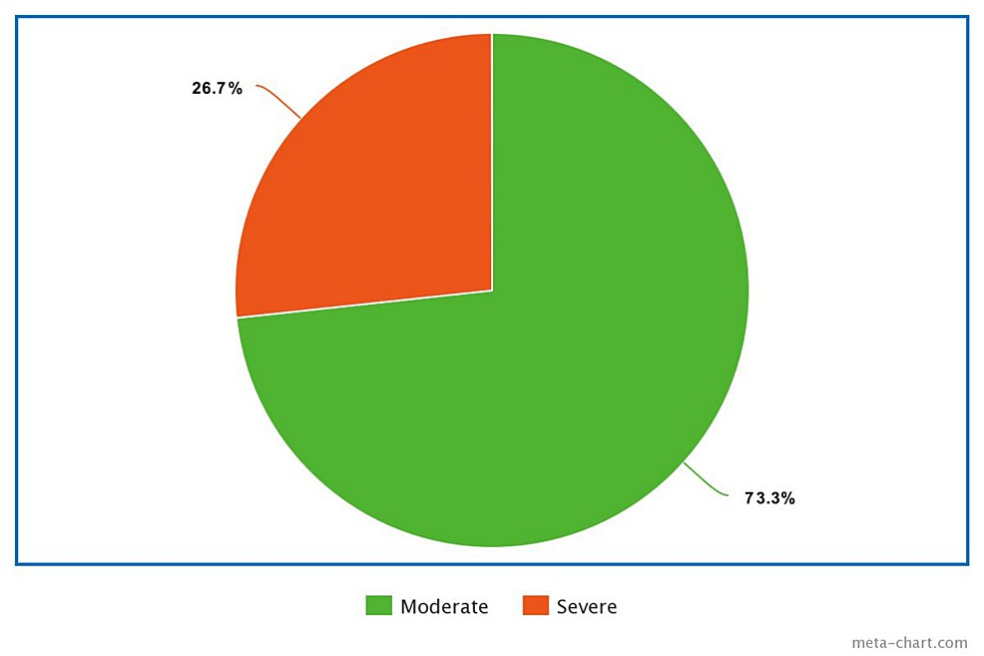
OHSS Severity Among the Enrolled Participants OHSS: ovarian hyperstimulation syndrome.

In terms of embryo transfers, three participants underwent three transfers and 12 underwent two transfers (Figure 4). The data provided on the severity of ovarian hyperstimulation syndrome (OHSS) indicated that 26.7% of the participants experienced moderate OHSS, while the majority (73.3%) were severe OHSS (Figure 5).

**Figure 6:**
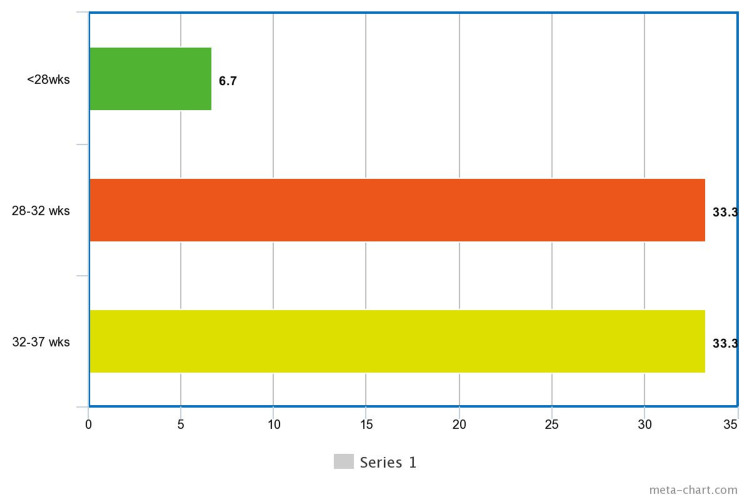
Preterm Delivery Distribution Among the Enrolled Participants

**Figure 7:**
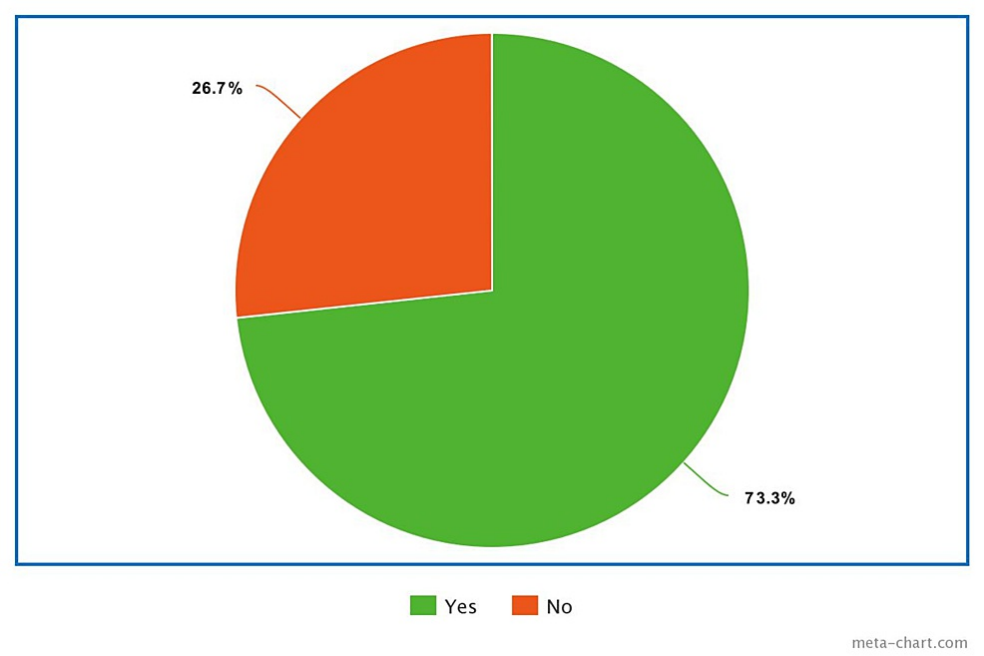
Pregnancy Rate Among the Enrolled Participants

Preterm delivery cases were further analyzed to identify whether preterm delivery as an effect of assisted reproductive technology (ART) cycle is complicated by OHSS. Among the participants, 33.3% delivered between 32 and 37 weeks, 6.7% before 28 weeks, and 33.3% delivered within the 28-32 week range (Figure 6). The occurrence of pregnancy among the participants showed that 73.3% experienced pregnancy, while the remaining 26.7% ended by abortion (Figure 7).

**Figure 8:**
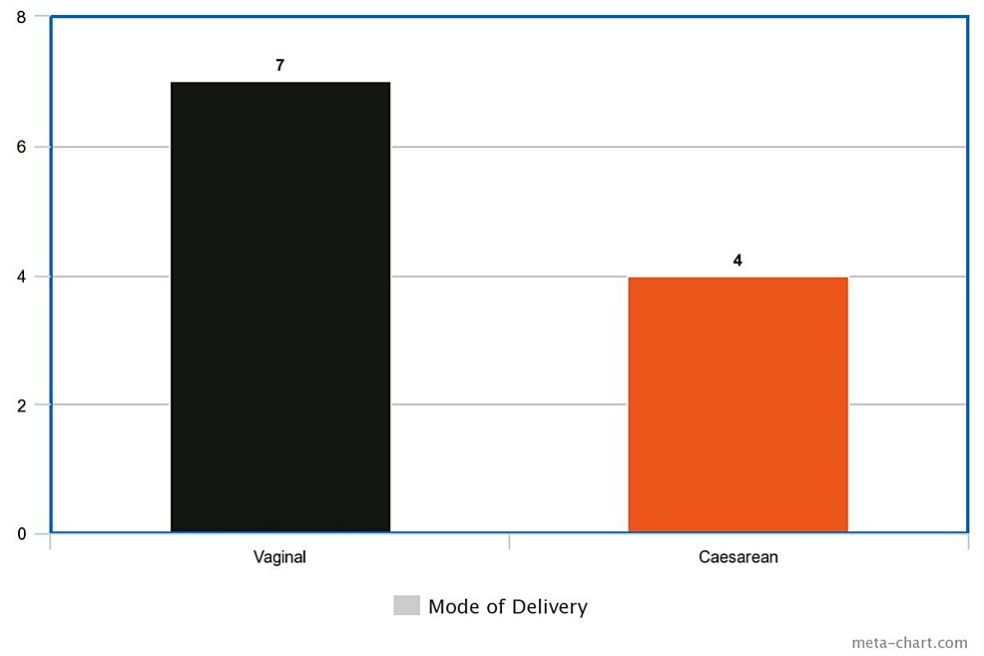
Mode of Delivery

Finally, data regarding the mode of delivery indicated that seven participants gave birth vaginally, and four had a cesarean section birth (Figure 8).

## Discussion

As we delve into the discussion of our findings, it is crucial to reiterate the context and objectives of our study. We embarked on this research journey to explore the pregnancy outcomes of women undergoing assisted reproductive technology (ART) procedures, particularly those complicated by ovarian hyperstimulation syndrome (OHSS). This case series study aimed to provide insights into the prevalence, implications, and management of OHSS in the context of ART and to identify areas requiring further investigation. Our examination encompassed several key pregnancy outcomes including successful pregnancies, instances of abortions, rates of preterm deliveries, and the mode of delivery. As we move forward in this discussion, we will unpack our findings, draw connections to the existing research, identify the implications of our study, and suggest directions for future research.

Our case series analyzed the outcomes of pregnancies from assisted reproductive technology (ART) cycles which are complicated by ovarian hyperstimulation syndrome (OHSS). From the sample of 15 participants, we observed that 26.7% had moderate OHSS, while the majority (73.3%) experienced severe OHSS. Notably, none of the participants were diagnosed with mild OHSS. When we evaluate the number of embryo transfers concerning the severity of OHSS, it was found that among those with moderate OHSS, two embryo transfers were more common, while those with severe OHSS more frequently underwent three embryo transfers.

In assessing the types of infertility, primary and secondary infertility were equally represented in our sample. Anovulation was found to be the most common factor contributing to infertility. Regarding pregnancy outcomes, there was a mix of preterm and term deliveries, with a noteworthy proportion delivering before the completion of the full term. The distribution of pregnancy rates was uneven, and the mode of delivery varied with both vaginal and cesarean options represented. Additionally, a fraction of the participants experienced a termination of their current pregnancy.

Interestingly, only one of the participants developed gestational diabetes mellitus (DM) during their pregnancy. This suggests that women with a history of infertility undergoing ART cycles complicated by OHSS may not have a risk of developing gestational DM. Yet, to validate this, further studies with a larger sample size are required. Similar studies have examined the pregnancy outcomes of ART cycles, with OHSS being a central consideration. For instance, Bourdon et al.'s meta-analysis of 36 studies found a higher risk of preterm delivery and cesarean section in cases of OHSS, mirroring the findings of our case series [12]. On the other hand, Capuzzo et al.'s work suggests that OHSS could be associated with lower pregnancy rates [13].

The suggestion by Capuzzo et al. that OHSS could be associated with lower pregnancy rates may be underpinned by several physiological factors related to the condition [13]. OHSS, characterized by an over-response to ovary-stimulating drugs, can cause ovarian enlargement and a shift in bodily fluids into the third space (such as the abdominal cavity). This result contradicts our findings, although our smaller sample size may be a factor here.

Ma et al.'s study linked OHSS with an increased risk of gestational diabetes and preeclampsia [14]. In contrast, our case series did not observe any cases of these conditions, except for one participant with elevated blood sugar levels hinting at a possible risk of gestational diabetes [14]. It should be emphasized again that the smaller sample size in our case series could influence the results. In terms of preterm delivery rates, our case series reported a higher incidence than some previous studies, such as the works by Rosario et al. [15] and Dobrosavljevic et al. [16]. It is important to note that these studies had larger sample sizes than ours, which could have led to different results.

Nonetheless, it is essential to acknowledge the limitations intrinsic to our case series. Firstly, due to the use of a case series design and non-probability purposive sampling, our findings might not be generalizable to a broader population of ART patients. Additionally, the relatively small sample size of 15 participants may restrict the broad applicability of our findings, as this may not fully represent the diverse range of clinical scenarios that could occur in larger or more diverse populations. Furthermore, our case series was limited to a single center, KAMC, which could introduce a bias, as the patient population, clinical practices, and outcomes could vary in different settings. The exclusion of certain patient groups, such as those with biochemical pregnancies, mild OHSS, or those who underwent PGT, may also limit the scope of our findings. Lastly, the use of retrospective data from patient's medical records could potentially introduce bias related to data accuracy and completeness. It is possible that relevant variables that were not recorded or were inaccurately recorded in the medical records could affect the results.

Despite these limitations, our case series provides initial insights that might guide future research. To validate and solidify our conclusions, larger-scale, multicenter studies with more diverse and representative samples are warranted. Indeed, considering the limitations of our case series, we may conclude: the data obtained in our study highlight the prevalence and potential severity of OHSS in ART cycles and its impact on various pregnancy outcomes such as pregnancy rates, abortion incidence, preterm deliveries, and modes of delivery. This underscores the need for more research to fully understand the relationship between OHSS and pregnancy outcomes, as well as to explore effective strategies to manage OHSS during ART procedures.

## Conclusions

In conclusion, our case series brings to light the intricate relationships between ART, OHSS, and various pregnancy outcomes within a specific group of women. While we found that OHSS in ART cycles may be associated with an increased risk of preterm delivery, cesarean section, and abortion, similar to the existing literature, our data did not suggest a clear link between OHSS and the development of gestational diabetes or preeclampsia. This diverges from some previous studies, potentially highlighting the complex and multifactorial nature of these conditions. Despite our study's limited sample size, our findings contribute to the broader understanding of OHSS and its impact on pregnancy outcomes in ART cycles, emphasizing the need for individualized patient care. Implications of our study include the potential for improved patient counseling, tailored treatment strategies, and the development of preventive measures against OHSS to enhance overall pregnancy success rates. Future research should build on these findings, further investigating the complexities of ART procedures complicated by OHSS, and exploring potential mitigations for these complications.
